# Increased short- and long-term mortality amongst patients with early periprosthetic knee joint infection

**DOI:** 10.1186/s12891-022-06024-y

**Published:** 2022-12-06

**Authors:** Olof Thompson, Annette W-Dahl, Anna Stefánsdóttir

**Affiliations:** 1 Department of Clinical Sciences Lund, Division of Infection Medicine, Lund University, Lund, Sweden; 2 Department of Infectious Diseases, Skåne University Hospital, Lund, Sweden; 3 Department of Clinical Sciences Lund, Division of Orthopedics, Lund University, Lund, Sweden; 4 Swedish Arthroplasty Register, Gothenburg, Sweden; 5 Department of Orthopedics, Skåne University Hospital, Lund, Sweden

**Keywords:** Periprosthetic joint infection, Knee arthroplasty, Mortality

## Abstract

**Background:**

Periprosthetic joint infection (PJI) following total knee arthroplasty (TKA) is a severe complication in terms of disability, morbidity, and cost. We performed a study to investigate whether early PJI (within 90 days of primary TKA) is associated with increased mortality. Secondary aims were to compare mortality rates over time and between surgical treatment methods.

**Methods:**

Patients with suspected PJI were identified by linkage of the Swedish Knee Arthroplasty Register (SKAR) and the Swedish Prescribed Drug Register (SPDR) in 2007–2008 and 2012–2013. Medical records of patients receiving more than 4 weeks of continuous antibiotic therapy were subsequently reviewed to verify the PJI diagnosis. Information on mortality was obtained through the SKAR which is updated daily from the tax agency and patients with PJI were compared to patients without PJI.

**Results:**

Four hundred sixty-six patients were diagnosed with PJI within 90 days and compared to 40,362 patients without PJI. Mortality rates were significantly higher for PJI patients in both short- and long term: 2.6% vs. 0.8% at 1 year, 4.9% vs. 1.9% at 2 years, 15.7% vs. 7.1% at 5 years, and 38% vs. 21.4% at 10 years. The difference in mortality rate remained after adjusting for sex, age, diagnosis, and time period for surgery with Hazard Ratio 1.8 (95% CI:1.6–2.1). Mortality rates did not differ between time periods, and we found no correlation to surgical treatment.

**Conclusion:**

Patients with early PJI after primary TKA have an increased mortality rate compared to TKA patients without PJI. Improvements in surgical treatment strategy has not resulted in better survival. Long term difference in mortality rates indicates that PJI is not the sole reason for mortality suggesting a general frailty in PJI patients.

## Introduction

Total knee arthroplasty (TKA) is an increasingly common procedure worldwide, and numbers are expected to rise further in years to come [1]. The most frequent cause for revision surgery within 2 years of implantation is periprosthetic joint infection (PJI), with a reported 2-year incidence of 1.44% in Sweden [2]. PJI impacts individual patients and the health-care system with additional surgical procedures, hospitalizations, and long courses of antimicrobials as well as worse functional outcomes and increased health-care costs [3, 4]. In addition, PJI has been associated with increased mortality in both short- and long-term [5, 6], highlighting the need for preventive measures [7].

Treatment practices for PJI have evolved in the last 2 decades and have been more consistent in the last 10 years, due to development of national and international definitions, treatment guidelines and consensus documents [8–10].

Previous investigations on PJI associated mortality usually emanate from single centers and/or mixing PJIs of the hip and knee. Few studies on mortality after PJI of the hip have recently been performed with data from large cohorts [6, 11], but large studies of mortality after PJI of the knee are scarce [12].

The aim of this study was to estimate the mortality rate of patients diagnosed with PJI within 90 days of primary TKA, and, more specifically, to ascertain whether patients with PJI had a higher mortality rate compared to patients without PJI. We hypothesized that improvements in treatment practices of PJI in later years would have a positive effect on mortality rates. A secondary aim was, therefore, to compare mortality rates between time periods and between surgical treatment methods.

## Patients and methods

### The Swedish knee arthroplasty register

The Swedish Knee Arthroplasty Register (SKAR, now a part of the Swedish Arthroplasty Register) holds data on primary- and revision arthroplasties collected from all public and private units performing arthroplasty in Sweden. The completeness is continually validated and is reported to be 97% in the studied time periods [13, 14]. The SKAR holds data regarding sex, age, fixation, diagnosis and, since 2009, ASA-class and BMI. The SKAR is updated daily from the tax agency with information on emigration and death. Patients are identified through personal registration numbers (PINs) which enables linking with other national registers as well as with local medical records.

### Study population

**Fig. 1:**
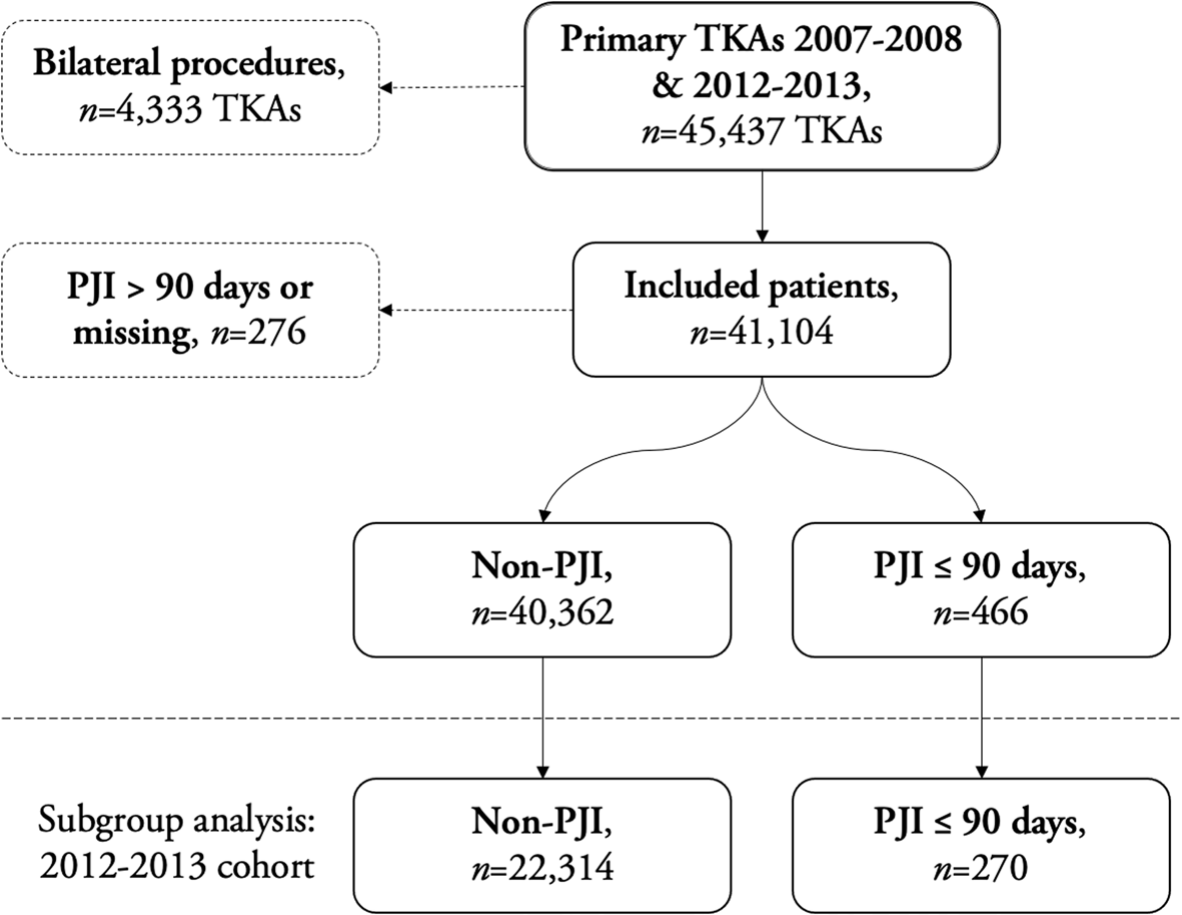
Flow chart of patient selection

All primary TKAs registered in the SKAR in 2007–2008 and 2012–2013 (*n* = 45,437) were included (Fig. 1). In a previous study [2] PINs were used to link the SKAR and the Swedish Prescribed Drug Register (SPDR) and identify patients with suspected PJI (*n* = 2505). The medical records were reviewed by local physicians to extract those with PJI (*n* = 644). The diagnosis was set by the treating physician, but uncertain cases were determined by the researchers. Retrospectively collected data included microbiological findings, inflammatory parameters, surgery, antimicrobial treatment, follow-up, and outcome. In patients with bilateral TKAs only the first TKA was included (*n* = 40,461), or in case of PJI, only the infected TKA. Survival data was extracted on October 1st, 2020. All patients were followed until death, emigration, or date of data extraction.

Patients diagnosed with PJI within the first 90 days after primary surgery (*n* = 466) were compared with patients without PJI (*n* = 40,384) in both time periods and in a subgroup analysis of the 2012–2013 cohort (*n* = 22,712). PJIs within 90 days were selected to capture predominantly patients with early postoperative infections, excluding most low-grade chronic and hematogenous PJIs. PJIs diagnosed between 91 days and 2 years were excluded from further analysis.

### Statistics

Descriptive statistics are presented as means (SD), medians (IQR) or numbers (%).

Incidence of mortality was assessed at 1-, 2-, 5- and 10-years using Kaplan-Meier analysis and visualized through Kaplan-Meier curves, using the Log-rank test to compare groups. Hazard ratios (HR) with 95% confidence intervals (CI) were calculated using Cox regression. We adjusted for the available confounders sex, age, diagnosis (dichotomized into osteoarthritis or not) and time period for primary surgery in the whole cohort. ASA-class and BMI became available in 2009 and subgroup analysis of the 2012–2013 cohort was therefore performed where ASA-class and BMI was included in the Cox regression model. ASA-class was dichotomized into ASA 1–2 and 3–4, respectively, and BMI was categorized into four levels: < 18.5 (underweight), 18.5–24.9 (normal), 25–29.9 (overweight) and ≥ 30 (obese). Proportional hazards assumption was assessed visually using a log (minus log) plot.

Mortality for PJI patients in the 2 time periods was compared using Kaplan-Meier analysis, limiting follow-up to 7 years.

### Ethics and funding

The study was approved by the local Ethics Review Board at Lund University on November 2, 2016 (Dnr 2016/28), and the need for informed consent was waived by the review board. Financial support to conduct the study was obtained through regional research grants and from Löf, the Swedish patient insurance.

## Results

A total of 41,104 patients were included in the analyses. 4333 patients had bilateral procedures in the study period and were, thus, only included once. In 466 patients a PJI was diagnosed within 90 days of primary surgery and in 40,362 patients no PJI was diagnosed. 276 patients with PJI after > 90 days or with missing information were excluded from further analysis (Fig. 1).

Patients with PJI within 90 days were more frequently male (58.4% vs. 40.7%), slightly older, and with a higher proportion of ASA-class 3–4. Indication for TKA was somewhat more often osteoarthritis in the non-PJI group (Table 1).

**Table 1:** Demographic and background variables for patients without PJI and patients with PJI diagnosis within 90 days of implantation

	Non-PJI		PJI ≤ 90 days	
	mean	SD	mean	SD
Age	69.2	9.1	70.1	9.5
BMI^a^	29.0	4.6	30.2	5.5
ASA^a^	1.98	0.60	2.2	0.60
	**n**	**%**	**n**	**%**
Total	40,384		466	
Sex, male	16,453	40.7	272	58.4
**ASA-class**^a^				
ASA 1–2	18,639	83.6	191	70.7
ASA 3–4	3647	16.4	79	29.3
**BMI-class**^a^				
< 18.5, underweight	41	0.2	0	0
18.5–24.9, normal	4125	18.5	45	16.7
25–29.9, overweight	9715	43.6	103	38.1
≥30, obese	8389	37.7	122	45.2
**Diagnosis**				
OA	39,024	96.6	433	92.9
Infl. joint dis.	873	2.2	21	4.5
Post traumatic	199	0.5	8	1.7
Other	288	0.7	4	0.9
**Fixation**				
Cementless	1193	3.0	11	2.4

^a^Variable available only for 2012–2013-cohort, *n* = 22,715. Numbers do not add up due to missing data

**Fig. 2:**
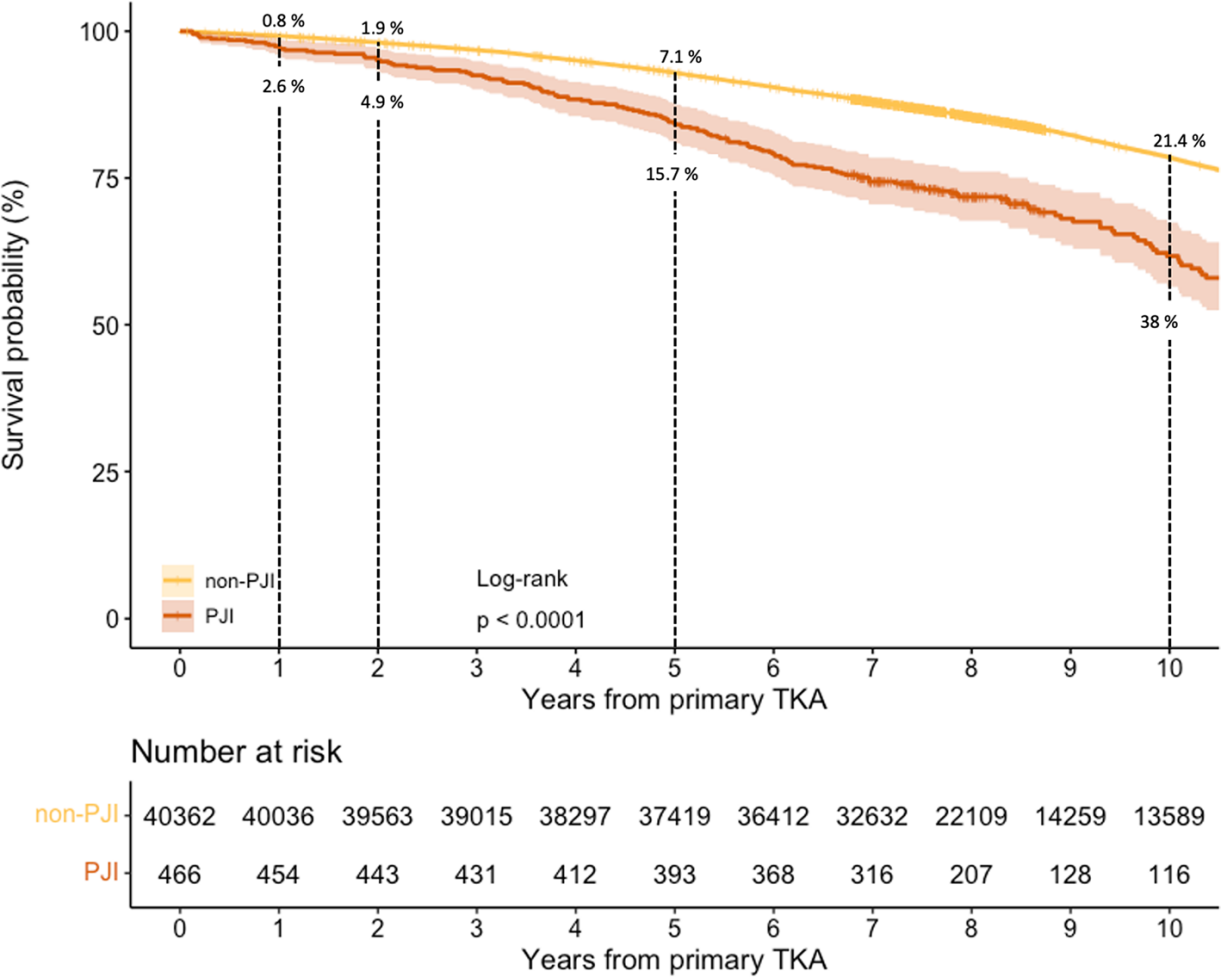
Kaplan-Meier survival curve comparing all-cause mortality for patients with PJI within 90 days of primary TKA and TKA patients without PJI. Data at 10 years available only for patients operated in 2007–2008. TKA, Total knee arthroplasty; PJI, Periprosthetic joint infection

The all-cause mortality rate was significantly higher in the PJI-group than in the group without PJI. Mortality rates were 2.6% vs. 0.8% at 1 year, 4.9% vs. 1.9% at 2 years and 15.7% vs. 7.1% at 5 years (Fig. 2.). Data for 10 years was available only for the first cohort. At 10 years the mortality rates were 38 and 21.4% for the PJI and non-PJI groups, respectively. The difference in mortality rate between groups remained after adjusting for sex, age, diagnosis, and time period for surgery, with HR 1.8 (95% CI:1.6–2.1).

In the subgroup analysis of the 2012–2013 cohort, ASA-class and BMI were available. Since ASA-class and BMI interact, BMI was not included in the final model. The increased risk of mortality for PJI patients remained (HR 1.93 [95% CI: 1.51–2.45]). Mortality in the ASA 3–4 group was increased (HR 2.02 [95% CI: 1.87–2.19]).

**Fig. 3:**
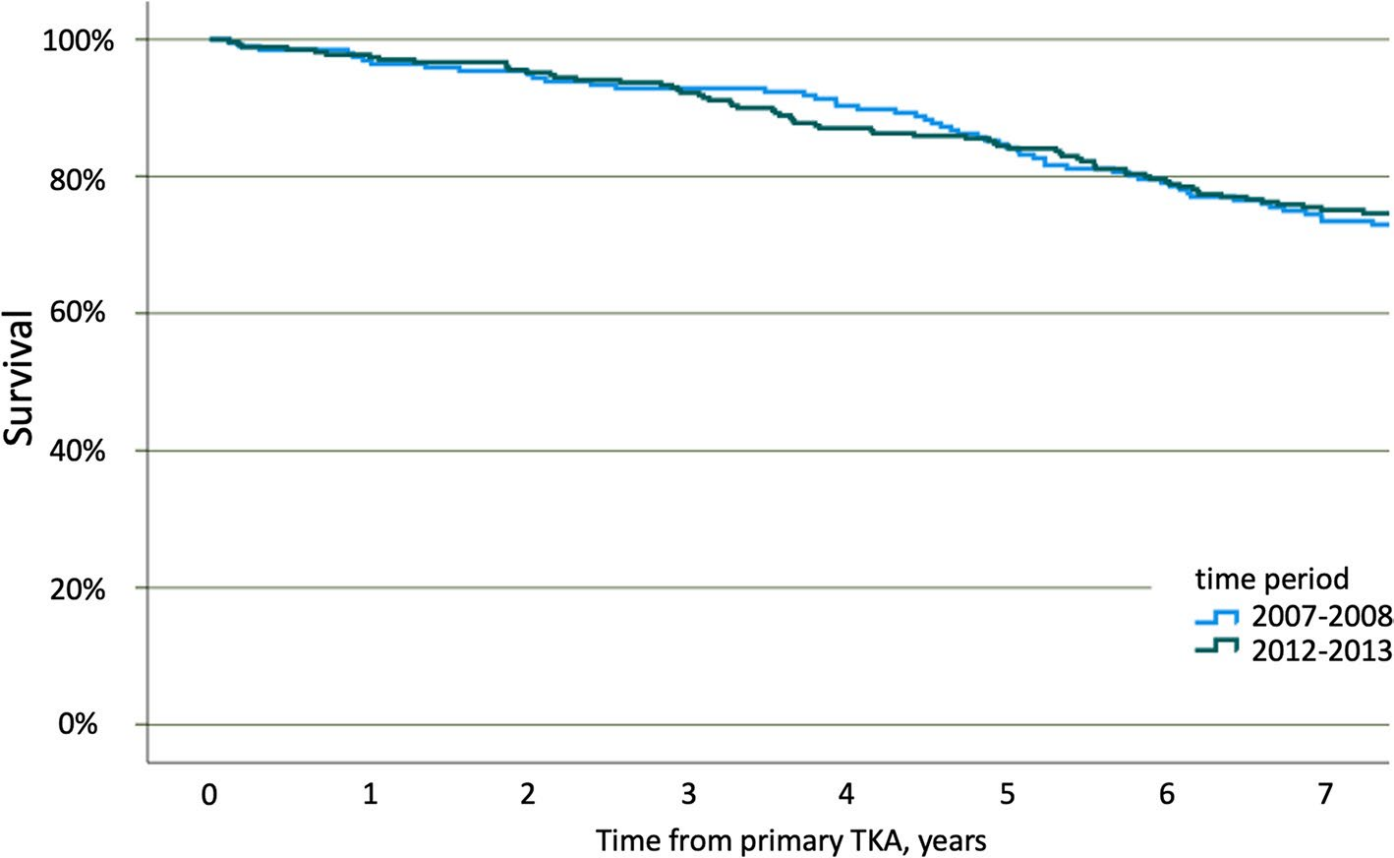
Kaplan-Meier survival curve comparing all-cause mortality for patients with PJI within 90 days of primary TKA during 2 time periods. TKA, Total knee arthroplasty; PJI, Periprosthetic joint infection

Over-all mortality was higher in the cohort operated in 2007–2008 (data not shown), but for patients with PJI within 90 days mortality was similar in the 2 time periods (Fig. 3).

**Fig. 4:**
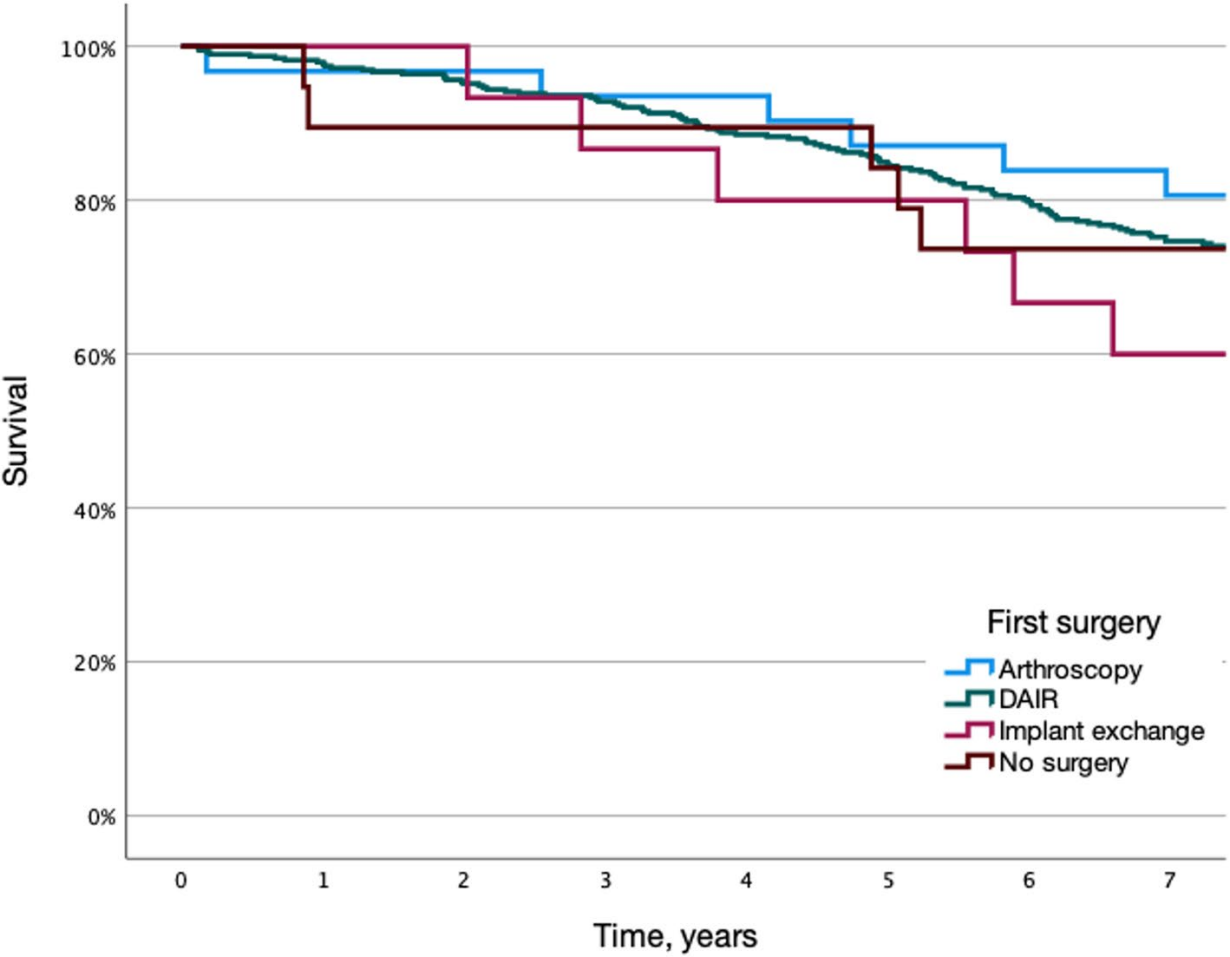
Kaplan-Meier survival curve comparing all-cause mortality for PJI-patients with different surgical treatment strategies. DAIR, Debridement, antibiotics, and implant retention

Details on time to diagnosis of PJI and surgical treatment are presented in Table 2. Treatment shifted towards an increased proportion of debridement with implant retention (DAIR) in the latter time period (76.7% vs 90.7%, *p* < 0.001). Mortality rates for the different treatment groups are illustrated in Fig. 4. We found no apparent associations between surgical treatment groups and difference in mortality rate. In the group that had more than one surgery a tendency towards increased mortality was seen when compared to patients that had only one surgery, but this difference was not statistically significant (log-rank test, *p* = 0.06). The difference in mortality between non-PJI and PJI patients remained after exclusion of PJI-patients that had more than one surgery (log-rank test, *p* < 0.001).

**Table 2:** Patients with PJI within 90 days of implantation in 2007–2008 and 2012–2013. Details of surgical treatment and time to diagnosis

	All		2007–2008		2012–2013	
	***n***	%	***n***	%	***n***	%
Arthroscopy	31	6.7	19	9.8	12	4.5
DAIR	392	84.1	148	76.7	244	90.7
Exchange	15	3.2	6	3.1	9	3.3
Arthrodesis, Resection, Amputation	3	0.6	3	1.6	0	0
No surgery	19	4.1	15	7.8	4	1.5
Unknown	2	0.4	2	1	0	0
One surgery	324	73	122	68.5	202	75.9
More than one surgery	120	27	56	31.5	64	24.1
Proportion diagnosed within 30 days	335	71.9	140	71.4	195	72.2
	**median**	**IQR**	**median**	**IQR**	**median**	**IQR**
Time to diagnosis, days	20	14–33	20	15–33	20	14–33
Time from diagnosis to surgery, days	1	0–2	1	0–2	1	0–2

## Discussion

We present an investigation on the association between PJI after primary TKA and mortality rate in a large, national cohort. We found a higher mortality rate after PJI compared to patients without PJI in both short- and long-term. The association remained after adjusting for available confounders such as age, sex, diagnosis, and, in the latter time period, ASA-class. We chose to analyze patients with PJIs diagnosed within 90 days of primary surgery to be able to capture predominantly early postoperative infections and to exclude the effect of hematogenous infections and secondary septic seeding as far as possible. More than 70% of the included PJIs were also diagnosed within the first 30 days (Table 2), strengthening this assumption.

The mortality rates at 1- and 5 years (2.6 and 15.7%, respectively) are somewhat lower than the 4.33 and 21.6% found in the meta-analysis by Lum et al. [5]. This could partly be explained by variations in patient selection. The meta-analysis by Lum et al. included a heterogenous sample of studies on two-stage revision knee-PJIs, whereas our material is comprised mainly of early PJIs that were treated with a DAIR strategy. Further, we included only primary TKAs, excluding patients with previous infection or revision for other causes. Since it is reasonable to assume that mortality is even higher in this group, it can explain some of the difference between the studies. No comparable nationwide studies on TKA exist to our knowledge, but some works on hip arthroplasty can be found. In the recent Swedish study on primary THA by Wildeman et al. a 10-year mortality rate after PJI in THA patients of 45% was found, somewhat higher than the 38% found in our study [6]. Similarly, Gundtoft et al. found a 1-year mortality rate of 8% following revision for THA PJI, quite a lot higher than the 2.6% in our study [11]. Hip fracture patients were included in both studies and is a group with known fragility. This may explain some of the difference, as the present study only included elective procedures.

As of 2018 the 10-year survival rate for all cancers in Sweden was 69% [15], suggesting a worse prognosis following PJI than for many cancers. We can only speculate on the reason for the increased mortality rate after PJI, since our investigation offers no data on causes of death. Some deaths can surely be attributed to the deep infections of themselves, but a majority probably have other causes. In our data 374 out of 466 PJIs (80%) were considered cured at the last follow up visit, suggesting a minor proportion of direct infection-related deaths. Further, when comparing cured infections with non-PJIs, a higher mortality rate was seen for the PJI cohort at 5- and 10-years (12.8 and 33.8%, respectively) but not at 1 year (0.5%) (data not shown). It has been recently proposed that PJI could be regarded as a chronic condition even after cure of the infection [6]. The decreased joint function and mobility would, according to this proposition, inhibit physical activity leading to a general health deterioration. Future research is needed to investigate this hypothesis. It is reasonable to assume that increases in mortality after PJI is not solely due to the infection, especially not more than 2 years after PJI diagnosis. Therefore, the presence of other conditions, predisposing for both PJI and death must be considered.

Previous studies identify several comorbid conditions that predispose patients to PJI [16–18], leading to a generally worse health in PJI patients than in arthroplasty patients in general. This is reflected in our data where PJI patients had slightly higher ASA-scores than the non-PJI cohort. ASA-score was included in the SKAR as a crude marker for comorbidity from 2009 and, though suffering from reliability issues, has been demonstrated to correlate to both increased mortality following arthroplasty and to increased risk for infection in other studies [16, 19]. Several comorbidities have also been identified as independent risk factors associated with increased risk of death within 1 year of PJI, such as diabetes, chronic lung disease and congestive heart failure [12]. These risk factors, if present, also affect the ASA-score. The effect of ASA-score on mortality in our study was therefore expected.

We found no visible difference in mortality between the 2 time periods in the study suggesting that improvements in treatment practice of PJI in latter years does not influence over all mortality in PJI patients. This is further elucidated by the fact that no significant difference in mortality was observed between different treatment modalities. In a previous study by Fischbacher implant exchange was associated with lower mortality than DAIR [20], a finding that has not been reproduced elsewhere. The choice of surgical treatment strategy is usually made depending on type of infection, status of the implant and surrounding tissue. It could be hypothesized that DAIR treated patients would have lower mortality since most patients undergo only one surgery. On the other hand, most DAIR treated patients have early infections that tend to be caused by more virulent bacteria than chronic infections that need implant exchange to be cured, possibly counteracting the benefits of fewer operations. It is of interest that the few patients that were not operated at all had a similar mortality rate as surgically treated patients, suggesting that infection eradication is not a prerequisite for survival.

The presented study has a few limitations. First, we used retrospective review of medical records to determine whether PJI was present or not, and diagnosis of PJI was determined by the treating physician. This leads to some variability, and PJIs may have been missed or falsely classified as infected. Further, there is an issue of immortality time bias in the PJI group that we were unable to control for. We believe the impact of this to be minor in the long term, with a maximum of 90 days increased survivorship in the PJI group. In the short term, however, mortality in the PJI cohort may have been underestimated due to this issue. Major risk factors for PJI and death, such as smoking and comorbidities, were unavailable to us. ASA score was included in the SKAR from 2009 and therefore only available for part of our cases. We included ASA score in our analyses where possible to try to ameliorate this lack. It would be interesting to investigate the cause of death in the PJI cohort, since this would elucidate the increased mortality rate further.

The major strength of our work is its size and nationwide setting. The use of the Swedish tax agency accounts for accurate mortality data with approximately zero data loss or misclassifications.

## Conclusion

Patients with PJI within 90 days after primary TKA have an increased mortality rate compared to patients undergoing primary TKA without PJI. Improvements in surgical treatment strategy has not resulted in better survival. Long term difference in mortality rates indicates that PJI is not the sole reason for mortality suggesting a general frailty in PJI patients.

## Data Availability

The datasets analyzed during the current study are not publicly available due to their use in ongoing research but are available from the corresponding author on reasonable request.
